# Low immunoglobulin levels increase the risk of severe hypogammaglobulinemia in granulomatosis with polyangiitis patients receiving rituximab

**DOI:** 10.1186/s12891-015-0860-3

**Published:** 2016-01-06

**Authors:** Emilio Besada

**Affiliations:** Bone and Joint Research Group, Institute of Clinical Medicine, UiT The Arctic University of Norway, 9037 Tromsø, Norway

**Keywords:** ANCA-associated vasculitis, Rituximab, Induction, Maintenance, Immunoglobulin, Hypogammaglobulinemia, Discontinuation, Adverse event, Principal component analysis, Correspondence analysis

## Abstract

**Background:**

Randomized controlled trials and retrospective studies in ANCA-associated vasculitis (AAV) concurred that rituximab (RTX) is effective to induce and maintain remission. Infections and hypogammaglobulinemia during RTX were usually infrequent and uncomplicated. But in the Tromsø study cohort, 45 % of patients with granulomatosis with polyangiitis (GPA) developed hypogammaglobulinemia during RTX maintenance leading to its discontinuation in 62 %.

**Methods:**

To explain these differences in outcome when using RTX in AAV to maintain remission, we used statistical structural methods to compare the Tromsø study cohort with other published cohorts.

**Results:**

GPA patients’ characteristics of the Tromsø study cohort were not so different compared with other cohorts. Rates of hypogammaglobulinemia and discontinuation of RTX seemed closely related to the cut-off used and to the levels of immunoglobulin (Ig) at baseline. Combination of low IgG serum levels at baseline (7.7 g/L) and low cut-off to define hypogammaglobulinemia in the Tromsø study cohort explained the high rate of hypogammaglobulinemia and discontinuation of RTX.

**Conclusions:**

Patients’ characteristics in the Tromsø study cohort were not skewed, apart from IgG levels. Low IgG level at baseline seemed to contribute the most to hypogammaglobulinemia and its complications.

## Background

Granulomatosis with polyangiitis (GPA) is an ANCA-associated vasculitis (AAV) affecting usually the small and medium vessels and is closely related to proteinase3-ANCA (PR3-ANCA). GPA often involves the upper and lower airways tracts and kidneys and was a fatal disease prior to the use of cyclophosphamide (CYC) [1].

Rituximab (RTX) is a chimeric monoclonal antibody against CD20 that depletes B cells [2] and is effective in inducing and maintaining remission in AAV patients in randomized controlled studies [3–5]. In two retrospective studies of remission maintenance with RTX, severe infections and hypogammaglobulinemia were frequent adverse events: 26–29 % had severe infections and 41–45 % had hypogammaglobulinemia [6–8]. However interpretation of the risk of hypogammaglobulinemia and its practical implications differed between the two studies. In the Tromsø study cohort, 17 % received intravenous immunoglobulin (IVIG) replacement and 62 % who developed hypogammaglobulinemia discontinued maintenance remission with RTX [8].

To explain differences in outcome, we compared using statistical structural methods the patients’ characteristics of the Tromsø study cohort with other published studies of induction and maintenance with RTX in AAV.

## Methods

### Identification of induction and maintenance studies

Two studies from the Northern Norwegian vasculitis register that was approved in 2001 by the Regional Ethical Committee for Medical and Health Research Ethics (REK-V 41/2001) were included [6, 8]. Patients (or the patients’ parents or guardians in case of children) gave written informed consent in accordance to the declaration of Helsinki at registry inclusion.

All other induction and maintenance studies with RTX in AAV were retrieved either from searching PubMed (www.ncbi.nlm.nih.gov/pubmed) in January 2015 using indexing terms ANCA vasculitis, rituximab maintenance and induction, or from reviewing the reference lists.

All studies of nine or more AAV patients were included in the analysis. Eighteen induction [3, 4, 6, 9–24] and 11 maintenance studies [5–8, 24–32] were identified. Definition of severe infections was similar in all studies, while definition of hypogammaglobulinemia differed.

### Statistical analysis

Principal component analysis (PCA) finds the best linear combinations of important variables at baseline. Number of patients, age, proportion of men, PR3-ANCA status, kidney and lung involvement, Birmingham vasculitis score (BVAS) at baseline, proportion of patients exposed to CYC and type of RTX induction regimen were included in the PCA of induction studies. Number of patients, age, PR3-ANCA status, kidney and lung involvement, exposure to CYC, disease duration prior RTX and follow-up in months were included in the PCA of maintenance studies. Correspondence analysis (CA) of adverse events during RTX maintenance included studies that reported the risks of both severe infections and hypogammaglobulinemia.

Statistical analysis was done with R (R project for statistical computing www.r-project.org).

## Results

### Induction

**Fig. 1:**
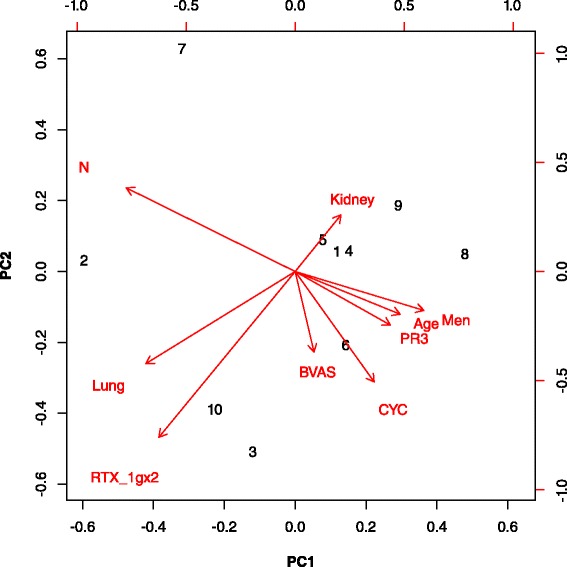
Principal component analysis of rituximab induction studies. 1: DE-Bad-Bramstedt [14]; 2: US-Cleveland [19]; 3: DE-Freiburg [24]; 4: DE-Munich [18]; 5: SE-Gothenburg; [15]; 6: SE-Stockholm [16]; 7: RAVE study [4]; 8: US-Rochester [11], 9: SE-Linköping [10]; 10: NO-Tromsø [6]. Age: age in years; BVAS: Birmingham Vasculitis Activity Score; CYC: proportion of patients exposed to cyclophosphamide; Kidney: proportion of patients with kidney involvement; Lung: proportion of patients with lung involvement; Men: proportion of men in studies; N: number of patients in studies; PR3: proportion of patients who are PR3-ANCA positive; RTX_1gx2: proportion of patients who received rituximab 1g twice given one fortnight apart (rheumatoid arthritis protocol)

Ten studies out of 18 were included in the PCA. Missing data from studies not included in the PCA were PR3-ANCA status [20, 21, 23], lung involvement [3, 22], CYC exposure [13, 17] and BVAS [12, 20] (Table 1 and Fig. 1). All studies concluded that RTX was effective in inducing remission in AAV patients, even though patients’ characteristics and induction regimen were different. The two first PCA dimensions explained 59 % of the variance of the cohorts’ characteristics included in the analysis.

**Table 1:** Characteristics of ANCA-associated vasculitis patients who received rituximab for remission induction

Study place	N	Men	Age	PR3-ANCA	Kidney	Lung	Orbital Subglottic	CYC exposed	Time to RTX	FU	RTX 1gx2	BVAS	IgG prior	IgG after	HypoG	SI	Ref
		%	y	%	%	%	%	%	mo	mo	%		g/L	g/L	%	%	
Linköping, Sweden	9	56	59	78	78	44	NA	100	36	12	0	6	8.4	6.4	NR	0	10
Rochester, US	10	70	57	100	70	40	10	100	59	9	0	6	NR	NR	NR	20	11
MC, UK	65	52	47	57	10	40	20	97	72	20	49	NR	7.9	8.0	0	20	12
Boston, US	39	49	60	62	NR	NR	NR	NR	67	18	90	1	NR	NR	0	3	13
Bad-Bramstedt Germany	59	59	54	86	44	41	46	100	37	7	0	11	8.6	6.9	12	26	14
Freiburg, Germany	37	57	62	81	60	81	NR	92	99	30	81	13	9.9	9.0	27	16	24
Goteborg, Sweden	29	52	51	97	62	59	38	100	31	21	0	6	NR	NR	NR	10	15
Stockholm, Sweden	16	56	60	81	50	50	NR	100	68	20	38	10	NR	NR	NR	38	16
London UK 2014	19	26	61	53	53	32	NR	NR	40	11.5	0	7	NR	NR	0	0	17
Munich, Germany	17	59	58	76	71	59	53	76	40	24	0	13	NR	NR	NR	24	18
Cleveland, US	105	48	49	75	58	83	NA	88	55	23	73	4	NR	NR	NR	7	19
London, UK 2009	10	50	49	NR	40	0	70	90	78	12	100	NR	NR	NR	NR	0	20
Torino, Italy	11	55	58	NR	64	27	NR	82	22	NR	0	23	8.0	6.7	NR	NR	21
London, UK 2011	23	52	59	70	100	NR	NR	100	1	36	100	21	NR	NR	4	9	22
Registry Germany	58	48	50	NR	45	59	19	60	54	18	47	13	NR	NR	NR	7	23
RAVE US	99	46	54	67	66	52	NR	42	30	6	0	9	NR	NR	NR	7	4
RITUXIVAS EUVAS	33	52	68	53	100	NR	NR	100	1	12	0	19	NR	NR	3	19	3
Tromsø, Norway	29	52	50	86	59	66	62	97	57	49	100	10	7.7	NR	NR	NR	8

*BVAS* Birmingham Vasculitis Activity Score, *CYC* cyclophosphamide, *FU* follow-up during maintenance remission with rituximab, *HypoG* rate of hypogammaglobulinemia, *MC* multicentric study, *N* number of patients, *NR* not reported, *RTX* rituximab, *Ref* reference, *SI* rate of severe infection

The RAVE and Cleveland Clinic studies recruited the most patients [4, 19]. The two 1g- RTX infusions given one fortnight apart were used more often at induction in the studies from Freiburg and Tromsø [8, 24].

Patients in the Tromsø cohort only had GPA, were more often PR3-ANCA positive, had more lung and less kidney involvement, received more frequently CYC and had a higher BVAS at baseline (Fig. 1).

### Maintenance

**Fig. 2:**
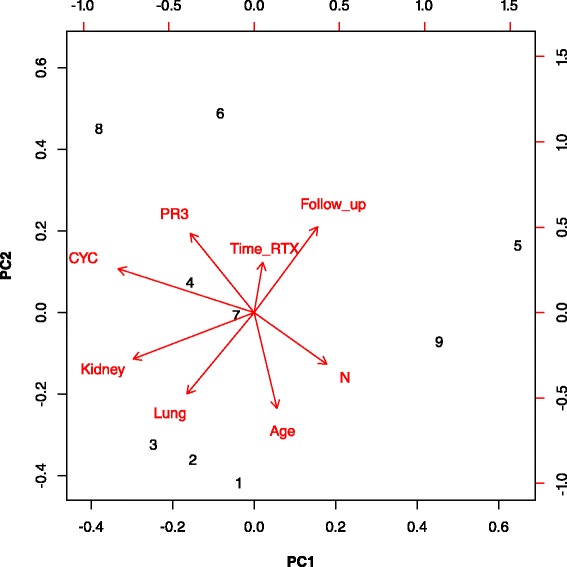
Principal component analysis of rituximab maintenance studies. 1: DE-Freiburg [24]; 2: FR-MC [32]; 3: FR-MAINRITSAN study [5]; 4: NO-Tromsø [6, 8]; 5: UK-Cambridge [7]; 6: US-Rochester [28]; 7: FR-Paris 2012 [26]; 8: UK-Nottingham [25]; 9: FR-Paris 2014 [30]. Age in years; CYC: proportion of patients exposed to cyclophosphamide; Follow_up: follow-up of remission maintenance with rituximab; Kidney: proportion of patients with kidney involvement; Lung: proportion of patients with lung involvement; N: number of patients included in the studies; PR3: proportion of patients with positive PR3-ANCA; Time_RTX: disease duration prior to rituximab

The PCA did not include two studies that reported neither age [31] nor exposure to CYC [29]. Studies were very different in term of patients’ characteristics at baseline, however all reported decreased disease activity and relapse rates during RTX maintenance (Table 2 and Fig. 2). The two first PCA dimensions explained 64 % of the variance of the cohorts’ characteristics included in the analysis.

**Table 2:** Characteristics of ANCA-associated vasculitis patients who received rituximab for remission maintenance

Study place	N	Age	Men	PR3-ANCA	CYC	CYC	RTX	Kidney	Lung	Orb-Subg	BVAS	Time to RTX	FU mo	IgG before	IgG after	SI	HypoG	Ref
		y	%	%	%	g	g	%	%	%		mo		g/L	g/l	%	%	
Nottingham, UK	11	41	64	100	100	NR	8	55	46	NR	14	72	32	NR	NR	NR	9	25
Paris 2011, France	28	51	60	68	100	48	NR	32	64	14	15	84	38	7.8	7.0	11	11	26
Rochester, US	53	46	47	98	100	NR	NR	42	49	NR	5	120	53	8.4	5.7	NR	NR	28
Freiburg, Germany	37	62	57	81	92	12	NR	60	81	NR	13	99	30	9.9	NR	16	27	24
Boston, US	172	60	45	43	NR	NR	NR	62	44	NR	2	NR	25	NR	NR	15	10	29
Paris 2014, France	66	50	49	80	89	29	4.6	21	47	14	10	67	34	8.3	7.5	14	2	30
Uppsala, Sweden	12	NR	42	100	100	61	NR	50	100	42	9	35	79	8.5	6.8	33	NR	31
MC, France	80	53	NR	84	98	13	NR	55	71	10	7	54	18	NR	NR	15	NR	32
Mainritsan, France	57	54	65	77	100	7.3	2.5	70	58	NR	NR	3	28	6.1	6.9	19	NR	5
Cambridge, UK	69	52	41	74	91	13.5	6	12	29	12	NR	60	59	9.3	8.0	29	41	7
Tromsø, Norway	29	50	52	86	97	17	9	59	66	62	10	57	49	7.7	4.9	24	45	8

*BVAS* Birmingham Vasculitis Activity Score, *CYC* cyclophosphamide, *FU* follow-up during maintenance remission with rituximab, *HypoG* rate of hypogammaglobulinemia, *MC* multicentric study, *N* number of patients, *NR* not reported, *Orb-Subg* frequency of orbital-subglottic involvement, *RTX* rituximab, *Ref* reference, *SI* rate of severe infection

Studies from Freiburg [24] and two other multicentric French studies [5, 32] were closely related, as their patients were older at RTX induction, had more kidney and lung involvement and had the shortest follow-up after RTX. Almost all patients were PR3-ANCA positive in the studies from Mayo Clinic and Nottingham [25, 28]. Cohorts from Tromsø and Paris were closest to the centroid (point 0) [8, 26].

Patients in the Tromsø cohort only had GPA, were younger, more often PR3-ANCA positive and received more frequently CYC prior to RTX (Fig. 2).

### Severe infection and hypogammaglobulinemia during maintenance

**Fig. 3:**
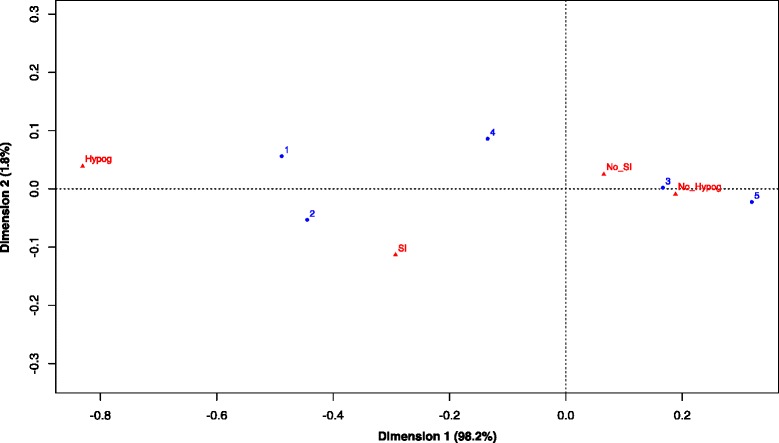
Correspondence analysis of adverse events in studies on RTX maintenance in AAV. Hypog: hypogammaglobulinemia; No_Hypog: absence of hypogammaglobulinemia; No_SI: absence of severe infection; SI: severe infection. 1: Tromsø Norway [6, 8]; 2: Cambridge UK [7]; 3: Boston USA [29]; 4: Freiburg Germany [24]; Paris France [30]

A total of 18 % (range 11–33 %) of patients had severe infections during RTX maintenance [5–7, 24, 29–32]; 18 % (2 – 45 %) developed hypogammaglobulinemia [7, 8, 24, 25, 29, 30]. The CA included five cohorts [6–8, 24, 29, 30] as data of both severe infection and hypogammaglobulinemia were lacking in most studies. The Tromsø and Cambridge study cohorts had more severe infection and hypogammaglobulinemia [6–8] compared with Freiburg, Paris and Boston [24, 29, 30] (Fig. 3). Risks of severe infections and hypogammaglobulinemia were equivalent in the studies of Cambridge and Tromsø [6–8]. While severe hypogammaglobulinemia occurred in 7 % of the patients in the Cambridge cohort [7], hypogammaglobulinemia led to RTX discontinuation in 62 % and 53 % in the Tromsø and Boston cohorts [8, 29].

Ig levels were lower in patients from the Tromsø study cohort both at baseline and during RTX maintenance: IgG 7.7 compared with > 8.3 g/L at baseline in the other studies [7, 24, 29, 30] and IgG 4.9 during maintenance compared with > 7.0 in Boston [29], 7.5 in Paris [30], 8.0 in Cambridge [7] and 9.0 in Freiburg [24].

## Discussion

The Tromsø study cohort only included GPA patients; they were more frequently PR3-ANCA positive and were often exposed to CYC compared with other cohorts. Patients’ characteristics of the Tromsø cohort were similar to Freiburg during induction [24] and to an older cohort from Paris during maintenance [26]. The Tromsø study cohort seemed representative of maintenance studies since it was close to the centroid of the PCA.

In AAV, the risks of hypogammaglobulinemia and of severe infection seemed equivalent at 18 % during remission maintenance with RTX. While severe infection was defined alike in all studies, the definition of hypogammaglobulinemia was not standardized and identified patients with different Ig serum levels conferring different risk to receive IVIG and to discontinue RTX. Hypogammaglobulinemia cutoff was lowest in the Boston study: total IgG < 4 g/L [29] vs. IgG < 6 in Cambridge [7], IgG < 7 [24] in Freiburg, total Ig < 6 (corresponding to IgG < 5 g/L) in Tromsø [8] and total Ig < 7 g/L in Paris [30]. Difference in cutoffs explained the difference in results between studies, but hypogammaglobulinemia in the Tromsø study was still more frequent and severe.

Fifty three and 62 % discontinued RTX maintenance due to hypogammaglobulinemia in the Boston and Tromsø cohorts [8, 29]. However the risk of hypogammaglobulinemia was four times higher in the Tromsø cohort since the Boston study used a lower cutoff to define hypogammaglobulinemia [29].

The Cambridge and Tromsø studies had an increased risk of hypogammaglobulinemia, respectively 41–45 % [6–8]. However the impact of hypogammaglobulinemia was more severe in the Tromsø study. In the Tromsø study, patients had lower IgG levels during RTX maintenance and 17 % required intravenous immunoglobulin (IVIG) replacement [8]. Only 7 % required IVIG in the Cambridge study [7].

PR3-ANCA status and exposure to CYC prior to RTX were similar in the Tromsø and Cambridge cohorts. Nevertheless others factors, such as organ involvement and treatment strategy during remission maintenance, could also explain the differences in severity of hypogammaglobulinemia between both studies. GPA patients in the Tromsø study had more lung and kidney involvement than in Cambridge. They also used concomitant immunosuppressive drugs for a median of two years and continued with pre-emptive RTX maintenance for a median of four years. While in the Cambridge study, patients did not use concomitant immunosuppressive drugs, received RTX remission maintenance during two years and were only re-treated with RTX in case of relapse [7].

## Conclusions

In summary, GPA patients’ characteristics in the Tromsø cohort were not skewed compared with the other cohorts, apart from IgG levels. In agreement with a previous study on RTX use in autoimmune diseases [33], low IgG level at baseline seemed to contribute the most to hypogammaglobulinemia and its complications during RTX maintenance in AAV.

### Ethics approval and consent to participate

Human data originated from the Northern Norwegian vasculitis register that was approved in 2001 by the Regional Ethical Committee for Medical and Health Research Ethics (REK-V 41/2001). Patients (or the patients’ parents or guardians in case of children) gave written informed consent in accordance to the declaration of Helsinki at registry inclusion.

## Abbreviations

AAV: ANCA-associated vasculitis
ANCA: antineutrophil cytoplasmic antibodies
BVAS: Birmingham vasculitis activity score
CA: correspondence analysis
CYC: cyclophosphamide
GPA: granulomatosis with polyangiitis
Ig: immunoglobulin
IVIG: intravenous immunoglobulin
PCA: principal component analysis
PR3: proteinase 3
RTX: rituximab
